# Alcohol Drinking Pattern: A Comparison between HIV-Infected Patients and Individuals from the General Population

**DOI:** 10.1371/journal.pone.0158535

**Published:** 2016-06-30

**Authors:** Maria Leticia R. Ikeda, Nemora T. Barcellos, Paulo R. Alencastro, Fernando H. Wolff, Leila B. Moreira, Miguel Gus, Ajacio B. M. Brandão, Flavio D. Fuchs, Sandra C. Fuchs

**Affiliations:** 1 Postgraduate Studies Program in Cardiology, School of Medicine, Universidade Federal do Rio Grande do Sul, R. Ramiro Barcelos 2600, 2°. andar, Porto Alegre, RS, Brazil; 2 Hospital Sanatório Partenon, State Department of Health, Rio Grande do Sul. Av. Bento Gonçalves, 3722, Porto Alegre, RS 90650–001, Brazil; 3 Postgraduate Program in Sciences in Gastroenterology and Hepatology, School of Medicine, Universidade Federal do Rio Grande do Sul, R. Ramiro Barcelos 2600, Porto Alegre, RS 90035–003, Brazil; 4 Postgraduate Program in Medicine-Hepatology, Universidade Federal de Ciências da Saúde de Porto Alegre, Porto Alegre, Brazil; 5 Cardiolody Division, Hospital de Clinicas de Porto Alegre. Centro de Pesquisa Clínica, R. Ramiro Barcelos 2350, Porto Alegre, RS 90035–003, Brazil; University of Missouri-Kansas City, UNITED STATES

## Abstract

**Background:**

Alcohol consumption is highly prevalent in the general population and among HIV-infected population. This study aimed to compare the pattern of alcohol consumption and to describe characteristics associated with heavy alcohol consumption in individuals from the general population with patients infected with HIV.

**Methods:**

Participants for this analysis came from a population-based cross-sectional study and from a consecutive sampling of patients infected with HIV. Participants aged 18 years or older were interviewed using similar questionnaires with questions pertaining to socio-demographic characteristics, alcohol consumption, smoking, physical activity, and HIV-related characteristics, among others. Blood pressure and anthropometric measures were measured using standardized procedures.

**Results:**

Weekly alcohol consumption was more prevalent among individuals from the general population than HIV-infected patients: 57.0 *vs*. 31.1%, P<0.001. The prevalence of heavy episodic drinking was higher in the population sample as well: 46.1 *vs*. 17.0%, P<0.001. In the general population, heavy alcohol consumption was more prevalent in men. Cigarette smoking was independently associated with heavy alcohol consumption among HIV infected (Prevalence Ratio; PR = 5.9; 95%CI 2.6–13.9; P<0,001) and general population (PR = 2.6; 95%CI 1.9–3.0; P<0.001). Years at school were inversely associated with heavy alcohol consumption among HIV-infected patients and directly associated among participants from the general population, even after controlling for sex, age, skin color, and smoking.

**Conclusions:**

Heavy alcohol consumption is more prevalent in the general population than among HIV-infected patients. Individuals aware about their disease may reduce the amount of alcoholic beverages consumption comparatively to healthy individuals from the general population.

## Introduction

Regular consumption of alcoholic beverages is a habit of at least half of the world adult population [1–4]. This habit decreases with age, is more common among men and is directly associated with socioeconomic status [1]. Heavy alcohol consumption occurs in 3 to 39% of individuals [5–10]. The large variation in the prevalence of heavy alcohol consumption may be partially explained by the variable definition of heavy alcohol consumption [7,11].

Diseases related to alcohol consumption vary in frequency and by region. Eastern Europe and Latin America have the highest burden of mortality attributable to alcohol consumption, which primarily affects men [12]. The risk is directly related to the amount of alcohol consumed [11]. People infected with HIV also have heavy alcohol consumption, ranging from 8 [13, 14] to 50% [15–17], which may result in lower adherence to antiretroviral treatment (ART), reduced immunity [18], and progression to AIDS [18–21]. Besides, alcohol consumption may increase risky sexual behavior, such as unprotected sex, multiple partners, and selling sex [22]. In addition, few individuals with disorder associated with alcohol use receive treatment for HIV [23].

The possibility that the pattern of alcohol consumption differs between individuals in the population and in HIV-infected individuals is expected. The overall risky behavior of individuals infected with HIV may suggest that they abuse from alcoholic beverages more frequently [20]. On the other side, free-living individuals may consume higher amounts of alcoholic beverages because of beliefs of beneficial effects of alcohol in the prevention of cardiovascular disease [24,25]. Nonetheless, comparative studies of alcohol consumption in the general population and in patients infected with HIV have not been reported to date. Therefore, the aims of this investigation were to compare the prevalence of risky alcohol consumption and factors associated with this consumption in individuals selected from the population and individuals infected with HIV.

## Participants and Methods

### Study population

Two cross-sectional studies were conducted in Porto Alegre, southern Brazil, to investigate alcohol consumption among adults aged 18 years or older. The population-based study [26,27] and the study of risk factors for metabolic syndrome and lipodystrophy in HIV-infected patients [28] used similar methods and instruments to investigate risk factors. In brief, the SOFT study enrolled 1858 participants, aged between 18 and 90 years, randomly selected through a multistage sampling. Participants were interviewed at home about risk factors for non-communicable diseases, cardiovascular morbidity and lifestyle [26,27]. In the SMEL study, 1240 consecutive patients with 18 years or more, who were referred to an outpatient center for the treatment of HIV/AIDS were enrolled [28–30]. They were interviewed about use of antiretroviral treatment, morbidity associated with HIV-infection, lifestyle characteristics, risk factors for non-communicable diseases and cardiovascular disease [26,30]. Both studies were approved by the Institutional Review Board of the Hospital de Clínicas de Porto Alegre, which is accredited by the Office of Human Research Protections. All participants signed a consent form.

### Studied variables

Standardized questionnaires were used to investigate characteristics such as demographic (age, categorized into 18–34, 35–49 or ≥ 50 years; gender, and self-reported skin color: white or nonwhite), socioeconomic (education, defined by years at school, categorized into 0–4, 5–8 or 9–13 years), lifestyle (alcohol consumption and smoking), diabetes mellitus (previous medical diagnosis or use of anti-diabetic medication). The consumption of alcoholic beverages was estimated with questions about the amount and frequency of intake of each drink in the six months preceding the interview. The amount of ethanol, in grams per day, has been calculated and categorized into abstemious, social (0–15, for women, and 0–29, for men) or heavy (≥15, for women, and ≥30, for men [7, 27]. Heavy episodic drinking was defined by the consumption of five or more drinks on a single occasion. Individuals who used to drink several days during the week were considered weekly drinkers. Patients who smoked 100 or more cigarettes during lifetime were categorized as current smokers or former smokers, opposing to non-smokers. Participants have been interviewed regarding the HIV-infection and the use of antiretroviral therapy, and information was also obtained by the review of medical records. Data collection was performed by trained research assistants, under supervision, and 5% of interviews were repeated for quality control. Blood pressure was measured with an oscillometric monitor (OMRON CP-705), and the average of four measurements was employed in analysis. Diagnosis of hypertension was based on systolic blood pressure ≥140 mmHg or diastolic blood pressure ≥90 mmHg, or the use of blood pressure-lowering drugs.

### Sample size calculation and statistical analysis

The sample size calculation for the original studies was based on socioeconomic characteristics (such as, education level, measured by the years at school, SES) and risk factors for cardiovascular diseases or metabolic syndrome as outcomes [28,29]. The sample size of the SOFT study was able to detect a prevalence of heavy alcohol consumption of 20% among those with high SES and 15.0% among low SES, with 80% power and 95%CI, and to identify a prevalence ratio (PR) of at least 1.3. Data were entered in duplicate in a database created using EPINFO 7 (Centers for Disease Control and Prevention, Atlanta, United States). Statistical analysis was done with the Statistical Package for Social Sciences (version 17.0, SPSS Inc., Chicago, Illinois, USA) and the complex sampling module has been used in the population-based study. The Chi-square test and the t test or ANOVA were used in the bivariate analysis. Cox regression model, with time equal to one, was used to modelling the association of at risk drinking with sex, age, skin color, education, and smoking. Adjusted and unadjusted prevalence ratios (PR) and 95%CI were calculated.

## Results

We investigated 1858 in the population based survey and 1240 in the HIV-infected sample. Fifteen HIV-infected patients refused to participate and 40 did not meet the eligibility criteria due to age, deprivation of liberty or pregnancy. Table 1 shows characteristics of participants of the general population and HIV-infected patients. Individuals surveyed in the general population were, on average, older (43.9 ±19.1 *vs*. 39.1 ±10.1 years) and had higher education level (9.3 ±4.7 *vs*. 7.5 ±4.1 years at school) than the patients infected with HIV. Approximately a third of the general population and the HIV-infected were abstemious, and almost two thirds of both populations were current drinkers. Among the general population, 26% of participants were current smokers *vs*. 42.3% of HIV-infected, 66% of whom have used HAART during lifetime.

**Table 1:** Characteristics of subjects HIV-infected and the general population from southern Brazil [% or mean (SD)].

	HIV-infected (n = 1240)	General population (n = 1858)
Males	50.6	41.7
Age (years)	39.1 (10.1)	43.9 (19.1)
White skin color	57.3	71.6
Years at school	7.5 (4.1)	9.3 (4.7)
Current alcohol consumption	66.6	62.1
Smoking		
No	34.0	54.5
Ex-smokers	23.6	18.9
Current	42.3	26.6
HAART use	65.7	-
Diabetes mellitus	8.5	7.0
Hypertension	19.4	34.2

Weekly frequency of drinking was more common among individuals selected from the general population than among patients infected with HIV (57 *vs*. 31%; P<0.001) (Table 2). Heavy episodic drinking was also more frequent among individuals selected in the population (46.1 *vs*. 17.0%; P<0.001). Moreover, the prevalence of heavy alcohol consumption in the general population was almost twice the observed in the HIV-infected patients (10.3 *vs*. 5.6%; P<0,001). The analysis of current drinkers showed that about 92% of the general population sample and almost half of the HIV-infected population (46.7%) reported weekly intake of alcoholic beverages.

**Table 2:** Pattern of drinking among HIV-infected patients and the general population from southern Brazil (%).

	Overall		Current drinkers	
	HIV-infected (n = 1240)	General population (n = 1848)	HIV-infected (n = 826)	General population (n = 1027)
Weekly frequency of drinking	31.1	57.0	46.7	92.2
P value	<0.001		<0.001	
Consumption of alcohol				
Abstemious	33.4	37.9	-	-
Social	61.0	51.9	91.6	83.3
Heavy	5.6	10.3	8.4	16.7
P value	0.5		0.048	
Heavy consumption of alcohol	5.6	10.3	8.4	16.7
P value	<0.001		<0.001	
Heavy episodic drinking	17.0	46.1	25.4	77.2
P value	<0.001		<0.001	

Table 3 shows characteristics associated with heavy alcohol consumption in both studies with and without adjustment for sex, age, skin color, education, and smoking. The prevalence of heavy alcohol consumption was 78% higher in men from the general population than women. This association was observed in the HIV-infected population. The bivariate association between skin color and heavy alcohol consumption observed in the population sample was no longer significant after adjustment for confounding factors. There was no association among patients infected with HIV. Participants from the population sample and those infected with HIV had similar associations of other risk factors with heavy alcohol consumption. In the population infected with HIV, current smokers were approximately six times more likely to be heavy drinkers than non-smokers, while in the general population individuals who smoke were almost three times more likely. Heavy alcohol consumption was inversely associated with education in the HIV-infected population and direct associated in the general population. Among HIV-infected, participants who have hypertension were approximately two times more likely to be heavy alcohol drinkers than non-hypertensive participants, and the association became even more significant after controlling for confounding factors.

**Table 3:** Characteristics associated with heavy alcohol consumption among HIV-infected patients and individuals from the general population.

	HIV-infected		General population	
	PR (CI95%)	PR (CI95%)*	PR (CI95%)	PR (CI95%)*
Gender				
Women	1.00	1.00	1.00	1.00
Men	0.99 (0.98–1.00)	1.51 (0.92–2.50)	1.36 (0.95–1.94)	1.78 (1.25–2.53)
P	0.08	0.10	0.09	0.002
Age (years)				
18–34	1.00	1.00	1.00	1.00
35–49	0.69 (0.42–1.15)	0.62 (0.38–1.03)	0.73(0.48–1.13)	0.74 (0.48–1.16)
≥50	0.94 (0.48–1.83)	0.92 (0.48–1.80)	0.61(0.43–0.87)	0.76 (0.52–1.12)
P	0.3	0.16	0.03	0.3
Skin color				
White	1.00	1.00	1.00	1.00
Non-white	1.16 (0.73–1.83)	1.04 (0.65–1.66)	1.49 (1.03–2.15)	1.41 (0.99–2.02)
P	0.5	0.88	0.03	0.06
Years at school				
9–13	1.00	1.00	1.00	1.00
5–8	1.95 (1.04–3.66)	1.82 (0.95–3.45)	1.37 (0.98–1.91)	0.88 (0.61–1.27)
0–4	2.73 (1.45–5.12)	2.60 (1.30–5.15)	0.70 (0.37–1.31)	0.40 (0.25–0.72)
P	0.008	0.02	0.07	0.01
Smoking				
No	1.00	1.00	1.00	1.00
Ex-smokers	3.12 (1.20–8.15)	2.77 (1.04–7.38)	1.29 (0.86–1.94)	1.45 (0.94–2.25)
Current	6.70 (2.90–15.50)	5.96 (2.56–13.87)	2.62 (1.94–3.52)	2.61 (1.90–3.60)
P	< 0.001	< 0.001	<0.001	< 0.001
Diabetes mellitus				
No	1.00	1.00	1.00	1.00
Yes	0.86 (0.35–2.09)	0.98 (0.38–2.55)	0.80 (0.38–1.68)	0.61 (0.29–1.30)
P	0.7	0.9	0.6	0.2
Hypertension				
No	1.00	1.00	1.00	1.00
Yes	1.81 (1.11–2.97)	2.24 (1.32–3.80)	1.21 (0.87–1.69)	1.08 (0.75–1.56)
P	0.02	0.003	0.3	0.7

* Prevalence Ratio adjusted for sex, age, skin color, education, and smoking

## Discussion

This study found a lower prevalence of alcohol consumption on weekly basis, heavy episodic drinking, and heavy alcohol consumption in the population infected with HIV than among individuals from the general population. These differences were more pronounced among current drinkers. The association of risk factors with heavy alcohol consumption were not substantially different among participants from both samples, such as male gender, years at school, and smoking. Non-white skin color showed a borderline association in the general population, but no association was found among HIV-infected individuals. Hypertension was associated with heavy drinking in patients infected with HIV, but not among individuals of the population–based study. The prevalence of alcohol consumption observed in this study is in line with the estimates from other surveys, conducted in the general population and among HIV-infected patients [1–5, 31–33]. The prevalence of heavy alcohol consumption, however, was lower than previously described for HIV-infected individuals [13,14,16–17,21,33]. In a cohort study among U.S. veterans, 20% of those infected with HIV had a pattern of alcohol consumption that put them at risk for adverse health events and 33% were episodic heavy drinkers. Nonetheless, veterans were older, mostly males, and African Americans [17], while our sample had similar gender distribution and predominantly white skin color.

The novelty of our study is the finding that individuals infected with HIV have lower prevalence of risky patterns of alcohol use, such as weekly frequency, heavy consumption, and heavy episodic drinking. We are not aware of a previous comparison of the consumption of alcohol in the general population and among HIV-infected individuals. The underlying reasons for lower prevalence of risky alcohol consumption by HIV-infected individuals may be related to their awareness of being sick, and the necessity to adhere to healthier life style recommendations to control their disease. They also may be concerned about the consequences of alcohol on the effect of antiretroviral drugs. On the other hand, individuals from the general population are probably less preoccupied with the deleterious effects of alcoholic beverages consumption [24,27,34]. They may even believe that alcohol is good for cardiovascular health. These hypotheses could not be tested in our study, since we did not investigate if participants of both studies had different expectations about the health effects of alcohol. Marked differences among current drinkers of the two populations are indirect evidence that HIV-infected patients have changed their pattern of drinking after the diagnosis of HIV. Population-based studies have shown an inverse association between excessive alcohol consumption and education [5–7,35–37], as well as a direct association [36–37] (an U shaped association). However the difference between those with high and low level of education seems to be the report of a drinking problem (negative consequences of alcohol), which is higher among those with less education, even when the pattern of alcohol consumption is similar [38]. In relation to the general population, our study showed results comparable to those observed in a British cohort [36] and a national survey in the U.S. [37]. In addition to the differences between populations and instruments used to characterize problematic alcohol consumption [23], some social attributes could explain the findings. For example, higher education level is associated with upper social status, occupations traditionally hold by men, that favor alcohol consumption, or also exposure to alcohol during college education [39]. On the other hand, the inverse association between education and heavy alcohol consumption has been reported for HIV-infected populations [33]. Moreover, less educated patients tend to have lower adherence to antiretroviral treatment [40,41] and to the recommendations for reducing alcohol consumption [36].

Smoking prevalence rate among the general population and patients infected with the HIV, detected in this study, were within the range reported in other studies [42,43,44]. This study also showed that prevalence was two to three times lower in the general population than among HIV-infected patients [42,45–47]. The increased prevalence ratio of heavy alcohol consumption among smokers from the two samples reproduces the association described in other surveys [48]. Beside the differences between general population and the HIV-infected regarding prevalence rate and risk factors for heavy alcohol consumption, there are some aspects that deserve to be mentioned. While consumption in low amounts is socially accepted and potentially protective for cardiovascular disease [24,49,50], in HIV-infected individuals this is not the case. All uses are harmful, either to the infection, progression to AIDS, interaction with antiretroviral drugs, or underlying HCV co-infection [23,51]. Abusive consumption of alcoholic beverages may reduce the care offered to patients infected with HIV [23].

Our study has limitations that should be mentioned, as the potential for recall bias. Nonetheless, the same questionnaire was used, as well as, the time interval to recall about alcoholic beverages consumption. The temporal difference between the data collection in the surveys (three years) may be another limitation of our study. There is evidence, however, from other population-based studies done in our city, that the pattern of alcoholic beverages consumptions remained relatively stable along the years [7,52]. Another potential limitation of our study is the fact that patients with HIV were interviewed in health care center and therefore may not reflect the overall HIV-infected population. Since in Brazil all patients with HIV must be registered to receive treatment free of charge, it is unlikely that they do not represent the entire population of patients with HIV.

## Conclusions

In conclusion, alcohol consumption is highly prevalent among HIV-infected patients and the general population. The lower prevalence of risky alcohol consumption in patients infected with HIV may be secondary to their concern about the harmful consequences over disease control, compared to the most liberal views about the risks of alcohol in the general population.

## Supporting Information

S1 Table Comparison of the pattern of alcohol drinking in men and women infected with HIV (%). (DOCX)
