# Supplementary material for: Alcohol Drinking Pattern: A Comparison between HIV-Infected Patients and Individuals from the General Population
Source: PLoS One. 2016 Jun 30;11(6):e0158535. doi: 10.1371/journal.pone.0158535 (PMC4928875; doi:10.1371/journal.pone.0158535)
Supplement: S1 Table — (DOCX) [file pone.0158535.s001.docx]

S1 Table. Comparison of the pattern of alcohol drinking in men and women infected with HIV [n (%)]

|  | Men (n=628) | Women (n=612) | P value |
| --- | --- | --- | --- |
|  |  |  |  |
| Weekly frequency of drinking | 250 (39.8) | 136 (22.2) | <0.001 |
| Consumption of alcohol |  |  | <0.001 |
| Abstemious | 161 (25.6) | 253 (41.3) |  |
| Social | 425 (67.7) | 332 (54.2) |  |
| Heavy | 42 (6.7) | 27 (4.4) |  |
| Heavy consumption of alcohol | 42 (6.7) | 27 (4.4) | 0.08 |
| Heavy episodic drinking | 144 (22.9) | 67 (10.9) | <0.001 |
